# The Pattern and Level of Knowledge on Obstetric and Newborn Danger Signs and Birth Preparedness among Pregnant Women in Dodoma Municipal: a Cross Sectional Study

**DOI:** 10.24248/eahrj.v4i1.624

**Published:** 2020-06-26

**Authors:** Theresia John Masoi, Stephen Mathew Kibusi, Alex Ernest Ibolinga, Athanase G. Lilungulu

**Affiliations:** a* Department of Nursing and midwifery, the University of Dodoma; a Department of Public Health, the University of Dodoma, Tanzania; b Department of Clinical Medicine, the University of Dodoma, Tanzania

## Abstract

**Background:**

Unacceptable high maternal mortality rates remain a major challenge in many low-income countries. Early detection and management of antenatal risk factors and good preparation for birth and emergencies are critical for improved maternal and infant outcomes. The aim of this study was to understand the pattern and level of knowledge on obstetric and newborn danger signs, Individual Birth Preparedness and Complication Readiness (IBPACR) among pregnant women in Dodoma Municipal.

**Methods:**

A quantitative cross sectional study was carried out between February and June 2018. A random selection of participants was employed to achieve a sample size of 450 pregnant women. A standard semi-structure questionnaire was used to collect data and descriptive analysis was carried out by using SPSS software to see the pattern and level of knowledge on obstetric danger signs and individual birth preparedness.

**Results:**

The mean age of participants was 25.6 years ranging from 16 to 48 years and majority 326 (72.4%) had 2 to 4 pregnancies. Only 203(45.1%) of the pregnant women were able to tell 8 and above danger signs with at least 1 from each of the 4 phases, with the most known obstetric danger signs being vagina bleeding during pregnancy 287(63.8), labour and delivery 234(52.0%), after delivery 278 (61.8) . 164 (36.4%) of the participants reported fever and difficult in feeding 182 (40.4%) as danger signs in newborn. Furthermore, only 75(16.7%) of the participants reported to be prepared for birth and complications. The most known component of birth preparedness was preparing important supply which are needed during birth 283 (62.9%).

**Conclusion:**

Results of this study showed a low level of knowledge on obstetric and newborn danger signs as well as poor individual birth preparedness and complication readiness. Important predictors of knowledge level and birth preparedness were found to be age, education level, gestation age at first visit and husband involvement in Antenatal visit and care.

## BACKGROUND

Identification and management of antenatal risk factors at early stage and good preparation are important for positive maternal and newborn.^1^

Despite of a number of Global and National efforts to improve women's health, the death of women during pregnancy, childbirth and after childbirth remains an unresolved challenge in many developing countries, including Tanzania.^2^ Almost 2 decades since the initiation of the Safe Motherhood Initiative, maternal mortality is still soaring high in many developing countries, about 830 women die from pregnancy or childbirth related complications around the world everyday.^3^

The maternal mortality ratio in developing countries in 2015 was 239 per 100 000 live births versus 12 per 100000 live births in developed countries. There are large disparities between countries, but also within countries and between women with high and low income and women living in rural areas versus women living in urban areas.^3^ The estimated Maternal Mortality Rate in the 2015-2016 Tanzania Demographic and Health Survey (TDHS- MIS) report was 556/100000 live births which is higher compared to the 2010 TDHS report which was 454/100000 live births.

Dodoma is among the regions with the highest maternal mortality rates in Tanzania. According to the 2012 census , Dodoma ranked the 9^th^ high burdened region with a maternal mortality rate of 512/100,000 live births.^4^

Maternal Mortality trends in Dodoma Region for the past 5 years were as follows: − 2012:60 deaths, − 2013:71 deaths, − 2014:69 deaths, − 2015:64 deaths and 2016:49. Trends of Perinatal death were: - 2012:599 deaths, − 201: 630 deaths, − 2014:715 deaths, − 2015:484 deaths, − 2016:517 deaths. The district with the highest number of maternal and perinatal deaths was Dodoma Municipal (Personal communication with the Dodoma Region Reproductive and Child Health Coordinator June, 2017)

In most developing countries, the underlying cause of maternal deaths during pregnancy and postpartum are attributed to 3 crucial delays; These include; (1) Identifying life threatening event danger signs and making the decision to go to the health facility (2) Delay in reaching the health facility and (3) Delay in receiving appropriate and adequate care at the health facility.^5^

Low knowledge about danger signs delays obstetric care seeking behaviours which thus contributes to high maternal mortality and morbidity worldwide.^6^ A study assessing determinants and awareness of danger signs and symptoms during pregnancy and complications among women in Jordan showed that 84.8% of the women interviewed were not aware of danger signs and symptoms of pregnancy complications.^7^

Another study was conducted in Chamwino District in Dodoma, Tanzania, the results showed that only 25.2% out of 428 respondents were knowledgeable about obstetric danger signs during pregnancy, childbirth and after childbirth.^8^ Studies in Tanzania have found that most women are not aware of danger signs of obstetric complications.^2^

Birth preparedness is a strategy to promote timely use of skilled maternal care especially during childbirth, based on the theory that preparing for childbirth reduces delays in obtaining this care. The proportion of preparing for birth and its complications has been found to be low in low-resource settings.^9^

A study on birth preparedness and complication readiness among recently delivered women in Chamwino showed that only 58.2 % of the respondents were considered as prepared for birth and its complications. The proportion of women prepared for birth and its complications was found to be low.^10^

Pregnant women and their families often ignore early warning signs due to lack of adequate knowledge and information about danger signs during pregnancy and labour and therefore delay in seeking health care services.^11^ Some of the factors that influence knowledge level and birth preparedness include; education level, parity, gravidity and age of a woman.

This study aimed at understanding the influencing factors on the level of knowledge o n obstetric and newborn danger signs, individual birth preparedness and complication readiness among pregnant women in Dodoma Municipal.

## METHODS

### Study Design

Descriptive cross-sectional study was conducted in Dodoma Municipal from February to June 2018, among pregnant women. Dodoma Region is 1 of Tanzania's 30 administrative regions and the location of the capital city of the country. It lies centrally in the eastern-central part of the country; it is about 300 miles (480 km) off the coast. Dodoma Urban District is 1 of the 7 districts of Dodoma region. It is bordered to the west by the Bahi district, and to the east by Chamwino District. According to the 2012 Tanzania National Census, the population of Dodoma Urban District was 410,956 covering an area of 2,576 square kilometres.^12^ Dodoma was one of the regions with the highest maternal mortality rates in Tanzania in 2012, Dodoma ranked the 9^th^ high burdened region with a maternal mortality rate of 512/100,000 live births.^4^ Within the municipal, there are 2 major Public Health facilities; the Makole Health Center which serves as the main antenatal care facility with an inpatient bed capacity of 55 beds and the Dodoma Regional Referral Hospital which is the highest level referral hospital in the region with an inpatient bed capacity of 420. In this study, pregnant women attending antenatal care and delivery services at Makole Health Centre, Chamwino Dispensary with the capacity of serving 100 pregnant women per day and 200 under 5 children per day and the Regional referral hospital were included in the study

**FIGURE 1.**
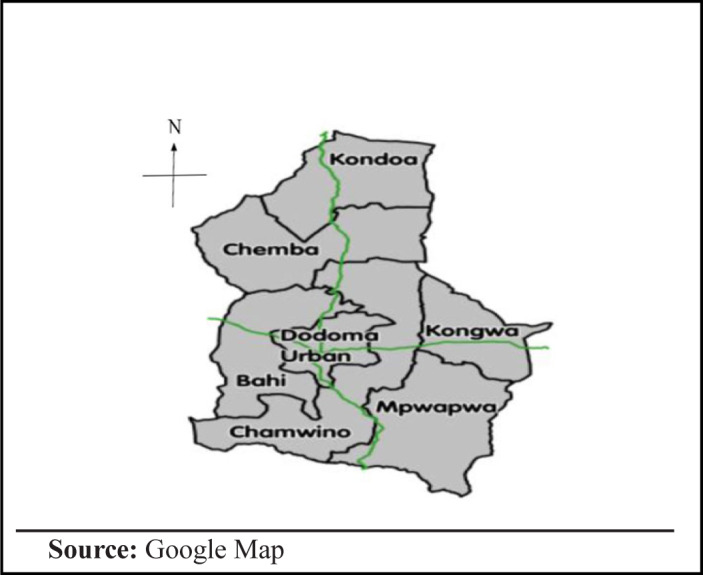
Dodoma Region Map

Source: Google Map

### Study Population

The study consisted of pregnant women attending Antenatal Care (ANC) visits in the health facilities of Dodoma Municipal

### Sampling Technique

A purposive sampling method was used to get Dodoma Region, Dodoma Municipal and the Health care facilities offering ANC and delivery care services in Dodoma Municipal. Participants were selected randomly. Participants who met the inclusion criteria and agreed to participate in the study on that particular day were listed together, where by every 3rd pregnant woman on that list was selected.

### Sample Size Calculation

The sample size was 450 pregnant mothers, which was obtained by using the following formula, and by using the Maternal Mortality ratio of Tanzania Demographic and Health Survey of 2015/2016, which was 556/100,000. Then;

$$\text{n}={\text{z}}^{2}\text{p}\left(1-\text{p}\right)/{\text{e}}^{2}$$

Where **n**= sample size

**z**= standard normal deviation of 1.96 corresponding to 95% confidence interval

**p**=proportion of the target population estimated using the 2015/2016 maternal mortality ratio for Tanzania; 556/100,000 live births.

$$\begin{array}{l} \text{e}=0.05.\\ \text{n}={\left(1.96\right)}^{2}*0.006\left(1-0.006\right)/{\left(0.05\right)}^{2} \end{array}$$

**n=439.94** minimum sample size, plus 5% Attrition An estimated sample of 450 pregnant women were included in the study.

#### Definitions of variables

- **Dependent variables**: Knowledge of obstetric and newborn danger signs, individual birth preparedness and complication readiness.
- **Independent variables**: parity, education level, age, gravidity and marital status.

#### Measurements of variables

- **Knowledge of the key obstetric danger signs (during pregnancy, childbirth, postpartum and in newborn) was scored as follows**;
  Those who did not mention any of the danger signs in all the 4 phases were considered to have no knowledge. Participants who mentioned up to 3 danger signs with at least 1 from each phase were considered to have low knowledge. Women who mentioned 4 to 7 danger signs with at least 1 from all the 4 phases were considered to have moderate knowledge of obstetric danger signs and the respondent was considered to be knowledgeable if she mentioned at least 8 danger signs. This method of scoring was adopted and modified from the study done in Southern Tanzania.^13^ This scoring method was again grouped into 2 groups of adequate knowledge and inadequate knowledge in the final analysis.
  The key danger signs in the 4 phases included:
  **Phase 1:** Danger signs during pregnancy (vaginal bleeding, swollen hands/face, severe headache, blurred vision, lower abdominal pain).
  **Phase 2:** Danger signs during labour/childbirth (severe vaginal bleeding, prolonged labour (>12 hours), convulsions, difficulty in breathing and retained placenta).
  **Phase 3**: Danger signs during postpartum (severe vaginal bleeding, foul-smelling vaginal discharge, and fever).
  **Phase 4:** Danger signs in the new-born; pitched cry, difficult feeding (unable to suckle), fits (convulsions), loss of consciousness, hot to touch (hyperthermia), difficult breathing, jaundice, failure to pass urine /stool in the first 24 hours.^8^
- **Individual birth preparedness and complications readiness was scored as follows**:
  Participants were asked to name items needed to prepare for birth and for emergencies. This was aimed at verify whether a participant was aware of the basic steps of Individual Birth Preparedness and Complication Readiness (IBPACR) i), Knowing Expected Date of Delivery (EDD) which was confirmed in her Reproductive and Child health 4 (RCH4) card, ii). Participants were also asked whether they had already identified a skilled birth attendant, iii) Identified the mode of transport for delivery and/or for obstetric emergency, iv) Saved money, v) Identified at least two blood donors, vi) Prepared supplies for birth and emergencies, vii) identified someone to escort them to labour, viii) Identified someone to take care of the family in her absence etc. Participants who scored 4 and above out of 9 basic steps were classified as having IBPACR while those who scored less than 4 were classified as “not having IBPACR. This scoring method has been previously used in studies which assessed women's level of birth preparedness and complications readiness at Chamwino District, Dodoma.^10^

### Data Collection

Semi-structured questionnaire with both closed and open-ended questions was developed to be interviewer-administered. This ensured that those unable to read and write could fully participate and also to ensure optimal capturing of all the needed information. The questionnaire included questions on socio-demographic characteristics, knowledge of key danger signs during pregnancy, childbirth, postpartum and danger signs in newborn, individual birth preparedness and complication readiness.

The questionnaires was first developed in English and then translated to Kiswahili which is the National language of Tanzania and the language used by the study population. The questionnaire was adopted from Jhpiego and modified to fit the Tanzanian context^14^ also from Tanzania Demographic and Health Survey 2015/2016 and from Nepal Demographic and Health Survey.^15^

### Data Analysis

In this study, data was analysed using the Statistical Product for Service Solutions (SPSS) software program version 21. Before conducting the analysis, error checking (data cleaning) was performed by using frequency distribution tables to see if all the data were entered correctly. Each variable was manually cross-checked to ensure validity and reliability of the findings. Scores that were out of range were corrected to avoid distortion of the statistical analysis. Descriptive analysis was used to analyse participant's characteristics to determine the frequencies and percentage of their distributions and also the pattern of level of knowledge and individual birth preparedness.

### Ethical Considerations

Permission to conduct this study was obtained from the University of Dodoma Research Committee. Ethical research clearance and research approval letters were obtained from the Graduate Office, University of Dodoma. Authorization to conduct the study in Dodoma Municipal and in the selected health facilities was obtained from Dodoma Urban District director and medical officer in charge of Human rights. Privacy, and Confidentiality were considered in this study. Research objectives, risk, and benefits of the study were well explained to the participants. Verbal and written consent were obtained from the participants and the questionnaires were answered voluntarily.

## RESULTS

### Social demographic and obstetric characteristics of the participants

A total of 450 pregnant women were included in the analysis, with a response rate of 100%. The mean age was 25.6 years (SD=6.1) with a minimum age of 16 years and maximum age of 48 years. As shown in Table 1 below, the most prominent age group n= 334(74.2%) ranged between 20 and 34 years.

**TABLE 1.** Social Demographic and Obstetric Characteristics of the Participants N=450

Variable		n(%)
**Age (years)**	< 20	71(15.8)
	20-34	334(74.2)
	≥ 35	45(10%)
**Education status**	Primary school	264(58.7)
	Secondary school	147(32.7)
	College/University	39(8.6)
**Occupational status**	Non-employed	144(32.0)
	Self-employed	298(66.2)
	Employed	8(1.8)
**Marital status**	Not married	97(21.6)
	Married	353(78.4)
**Gravidity**	1	97(21.6)
	2-4	326 (72.4)
	≥5	27(6.0)
**Parity**	1	121(26.9)
	2-4	309(68.7)
	≥5	20(4.4)
**Gestation age at first visit in weeks**		
	1-12 weeks	2(42.7)
	13-20 weeks	258(57.3)
**Age at first pregnancy in years**		
	<20yrs	214(47.4)
	20-34yrs	234(52.0)
	≥35 yrs	2(0.6)

On top of that, more than half of the participant, n= 264(58.6%) had primary school level of education and few with college/university education n=39(8.7%). Out of 450 respondents n=353(78.4%) were currently in marital union (married/ cohabiting). This study also explored the Obstetric characteristics among the study participants. As indicated below, out of 450 participants n=326(72.4%) had 2 to 4 pregnancies.

The minimum age of the respondent being pregnant at first was 14 years and maximum was 38 years with their mean age and standard deviation being 20 years(3.4). Other results are as shown in Table 1.

### Level of Knowledge on Obstetric and Newborn Danger Signs

Study participants were asked to mention obstetric and newborn danger signs which they know. It was found that overall score of the participants with adequate knowledge on obstetric and newborn danger signs was n=203 (45.1%) whereas 247 (54.9%) had inadequate knowledge. On knowledge level, individual scoring 8t and above with at least 1 from each of the 4 phases was regarded as having adequate knowledge and scored less termed as having inadequate knowledge.

### Level of Knowledge on Specific Key Obstetric and Newborn Danger Signs

The most known obstetric danger signs was vagina bleeding during pregnancy 287(63.8), labour and delivery 234(52.0%), after delivery 278 (61.8) and 164 (36.4%) of the study participants reported fever and difficult in feeding 182 (40.4%) as danger signs in newborn; other results are as shown in Table 2

**TABLE 2.** Scores of Knowledge on Specific Key Obstetric and Newborn Danger Signs

Key danger signs during pregnancy	n (%)	
	Yes	No
Vaginal bleeding	287 (63.8)	163 (36.2)
Swollen hands/face, ankle	131 (29.1)	319 (70.9)
Severe headache and blurred vision	56 (12.4)	394 (87.6)
Severe lower abdominal	152 (33.8)	298 (66.2)
Decreased or absent fetal Movements	52 (11.6)	398 (88.4)
Contractions/Labor pain before completed 37 weeks	31 (6.9)	419 (93.1)
**Key danger signs during labor/delivery**		
Severe vaginal bleeding	234 (52.0)	216 (48.0)
Prolonged labor (>12 hours)	56 (12.4)	394 (87.6)
Fits/Convulsions	38 (8.4)	412 (91.6)
Difficult breathing	39 (8.7)	411 (91.3)
Early rupture of membrane	35 (7.8)	415 (92.2)
**Key danger signs after childbirth**		
Severe vaginal bleeding	278 (61.8)	172 (38.2)
Foul-smelling vaginal discharge	148 (32.9)	302 (67.1)
Fever and convulsion	55 (12.2)	395 (87.8)
Placenta not delivered within one hour after delivery	38 (8.4)	412 (91.6)
Breast or nipple pain and fail to breastfeed	26 (5.8)	423 (94.0)
Severe headache and blurred vision	42 (9.3)	408 (90.7)
**Key danger signs in the newborn**		
High Pitched cry	179 (39.8)	271 (60.2)
Difficult feeding (unable to suckle)	182 (40.4)	268 (59.6)
Fits (convulsions) or loss of consciousness	73 (16.2)	377 (83.8)
Fever	164 (36.4)	286 (63.6)
Difficult breathing	39 (8.7)	411 (91.3)
Unable to pass urine and stool or both within 24 hours after delivery	19 (4.2)	431 (95.8)
Bleeding from the umbilical cord	54 (12.0)	395 (87.8)
Failure to cry immediately after birth	60 (13.3)	390 (86.7)
Jaundice/yellowish coloration	53 (11.8)	397 (88.2)

### Pattern and Level of Knowledge on Obstetric and Newborn Danger Signs within Different Categories

Pattern and level of knowledge differed within individual categories. Participants in the age group of 20 to 34 years were more knowledgeable on obstetric and newborn danger signs compared to other age groups. Also participants with college and university level of education scored higher than the lower levels of education. Other results are as shown in Table 3.

**TABLE 3.** Pattern and Level of Obstetric and Newborn Danger Signs within Different Categories N=450

Variable		Key danger signs	
		Inadequate knowledge n(%)	Adequate knowledge n(%)
**Age (years)**	< 20	45(63.4%)	26 (36.6)
	20-34	180(53.4%)	154(46.1%)
	≥ 35	22(48.9%)	23(51.1%)
**Education status**	Primary school	154 (58.3%)	110 (41.7%)
	Secondary school	73 (51.3%)	74 (48.7%)
	College/University	20 (49.7%)	19 (50.3)
**Accompanied by husband**	Yes	88(53.3%)	77(46.7%)
	No	159(55.8%)	126(44.2%)
**Gravidity**	1	101 (58.4%)	72 (41.6%)
	2-4	126(51.0%)	121 (49.0%)
	≥5	20 (66.7%)	10 (33.3%)
**Parity**	1	110(57.6%)	81 (42.4%)
	2-4	126 (52.5%)	114 (47.5%)
	≥5	11(57.9%)	8 (42.1%)
**Gestation age at first visit in weeks**			
	1-12 weeks	123 (54.4%)	103(45.6%)
	13-20 weeks	124 (55.4%)	100(44.6%)
**Age at first pregnancy in years**	<20yrs	109 (58.3%)	78 (41.7%)
	20-34yrs	138(52.9%)	123(47.1%)
	≥35 yrs	0 (0.0%)	2 (100.0%)

### Practices on Individual Birth Preparedness and Complication Readiness

Study participants were asked to tell how they have prepared themselves for birth and complications. It was found that only n=75 (16.7%) of the participants were able to tell at least 4 or more of the basic components of individual birth preparedness and complication readiness where as n=375 (83.3%) could not tell. On IBPACR score, participants who scored a total of 4 or more out of the 8 basic steps of IBPACR were classified as being prepared for birth.

### Knowledge of the Basic Components of IBPACR among the Study Participants

Participants were asked to tell the components of IBPACR ; the most known components by the study participants were saving money n= 277 (61.6%), Also most of the participants n=283 (62.9) said preparing important supply such as clothes as shown in Table 4.

**TABLE 4.** Knowledge on individual component of IBPACR

Basic IBPACR components	n (%)	
	Yes	No
know the expected date of delivery	169(37.6)	281(62.4)
Identify a skilled birth attendant	21(4.7)	281(62.4)
Identify the health facility which can be used in case of emergency or for childbirth	33(7.3)	417(92.7)
Identify 2 potential blood donors who would donate blood in case of Emergency	32(7.1)	418(92.9)
Preparing important supplies such as clothes needed for child birth	283(62.9)	167(37.1)
Identify a person who will escort to the health facility during an emergency	49 (10.9)	401(89.1)
Identify and arrange for transportation	143 (31.8)	307(68.2)
Saving money for delivery or for emergency	277 (61.6)	173(38.4)

### Pattern and Practice of IBPACR within Different Categories

As shown in Table 5; Results showed that Pattern and practice of individual birth preparedness was more observed among pregnant women who started their first ANC visit within the first 3 months of pregnancy, n=44 (19.5%) as compared to those who started late. On top of that, pregnant women who were accompanied by their husband to ANC clinics were more prepared compared to those who were not accompanied.

**TABLE 5.** Pattern and practice of IBPACR within different categories N=450

Variable		IBPACR categories IBPACR not prepared n(%)	IBPACR prepared n(%)
**Age (years)**	<20	54(86.7%)	17(13.3%)
	20-34	282(84.4%)	52(15.6%)
	≥ 35	39(76.1%)	6(23.9%)
**Education status**	Primary school	227(86.0%)	37(14.0%)
	Secondary school	121(82.3%)	26(17.7%)
	College/University	27(69.2%)	12(30.8%)
**Accompanied by husband**	Yes	134(81.2%)	31(18.8%)
	No	241(84.65%)	44(15.4%)
**Gravidity**	1	138(90.0%)	35(10.0%)
	2-4	210(85.0%)	37(15.0%)
	≥5	27(79.8%)	3(20.2%)
**Gestation age at first visit in week**			
	1-12 weeks	182 (80.5%)	44(19.5%)
	13-20 weeks	193 (86.2%)	31 (13.8%)

## DISCUSSION

Evaluating level of birth preparedness and complication readiness among pregnant women can also be measured by assessing knowledge of obstetrics danger signs.^14^ Having knowledge on obstetrics danger signs is an essential step in recognition of complications and enables one take appropriate action to access emergency care.^8^ Knowledge about danger signs among pregnant women is the key factors which influence timely access to care. The findings from this study showed a low level of obstetric and newborn danger signs but was higher compared to the study which was done in Ethiopia.^16^ The low knowledge on obstetric and newborn danger signs might be due to high proportion of pregnant women who had only primary level of education in this study. The most mentioned danger signs were vaginal bleeding during pregnancy, labour and delivery, severe vaginal bleeding after delivery and foul-smelling vaginal discharge, fever and convulsions as danger signs in newborn.

Education level and the number of pregnancies (gravidity) were good predictors of knowledge on danger signs. Participants with college/University level of education were more knowledgeable on key danger signs compared to those with lower education levels, these findings mirrors the findings of the study in Uganda on Obstetric danger signs and birth preparedness practices who also find education level to be an important factor.^17^ On top of that, participants with 2 to 4 number of pregnancies were also more knowledgeable compared to other categories.

Birth preparedness and complication readiness knowledge is derived from a combination of knowledge about obstetric and newborn danger signs. In this study, the prevalence of birth preparedness and complication readiness was estimated to be only 16.7%. This prevalence was found to be very low, similar to that of a study which was done in Mpwapwa district in Tanzania.^18^ Factors, such as education level and gestation age at first visit also played a greater role on birth preparation and complication readiness. Participants with college or university education level and those who start their initial ANC visit within the first 12 weeks of pregnancy were more prepared compared to other categories. These findings are similar with the study in Ethiopia on birth preparedness ^19^

All these factors could also be explaining the reasons for the high maternal mortality prevailing in Tanzania, because most of the women seem not to be prepared for birth and for emergencies and they are not much aware of the key obstetric and newborn danger signs that make them delay in making the decisions once problem arises.

## CONCLUSION

The study findings showed a low level of obstetric and newborn danger signs as well as individual birth preparedness among pregnant women in Dodoma Municipal. Level of knowledge differed among different age group, parity and gravidity. These findings reveal a need for innovative community-based educational strategies to increase the levels of knowledge about birth preparedness and complication readiness among pregnant women, which may help in minimizing and stop preventable maternal and newborn deaths. Also early booking for Antenatal care should be promoted at the community level, which may help in early identification and diagnosis of diseases and their related complication during pregnancy, labour and after delivery.

### Strength of the Study

The finding of this study showed a low level of knowledge on key danger signs and birth preparedness which provides an alert to the Government and other stakeholder on health related issue on where we are and where should we improve, as we still have high number of maternal death in Tanzania and specifically in Dodoma

### Study Limitations

The findings of the study could have been affected by the study setting, Dodoma Urban where the infrastructure are more improved compared to infrastructure in rural areas. Also, women in urban areas are more educated compared to those in rural areas, so these findings should not be generalised but instead more studies need to be done in these areas to come up with precise conclusions

## Abbreviations

ANC: - Antenatal care
MMR: - Maternal Mortality Ratio
MoHCDGEC: - Ministry of Health Community Development Gender, Elderly and Children
TDHS-MIS: - Tanzania Demographic and Health Survey-Malaria Indicator Survey
WHO: - World Health Organization
EDD: - Expected Date of Delivery
RCH4: - Reproductive and Child Health four
UDOM: - University of Dodoma
IBPACR: - Individual Birth Preparedness and Complication Readiness
NBS: -Nation Bureau of Statistics
